# Recognizing the emergency department’s role in oncologic care: a review of the literature on unplanned acute care

**DOI:** 10.1186/s44201-022-00007-4

**Published:** 2022-06-16

**Authors:** Rebecca S. Lash, Arthur S. Hong, Janice F. Bell, Sarah C. Reed, Nicholas Pettit

**Affiliations:** 1 Indiana University, School of Nursing, Liberal Arts 303b, 2101 E Coliseum Blvd, Fort Wayne, IN 46815 USA; 2 Division of General Internal Medicine and Department of Population and Data Sciences, University of Texas Southwestern Medical Center, 5323 Harry Hines Blvd, Dallas, TX 75390-6196 USA; 3 Betty Irene Moore School of Nursing, University of California, Davis, 2450 48th St #2638, Sacramento, CA 95817 USA; 4 California State University, Sacramento, 6000 J Street, Sacramento, CA 95819 USA; 5 Department of Emergency Medicine, Indiana University School of Medicine, 720 Eskenazi Ave, 3rd Floor FOB, Emergency Medicine, Indianapolis, IN 46202 USA

**Keywords:** Oncology, Cancer, Emergency, Emergency department, ED, ER, Use, Utilization, Visit, Visits

## Abstract

**Background:**

The global prevalence of cancer is rapidly increasing and will increase the acute care needs of patients with cancer, including emergency department (ED) care. Patients with cancer present to the ED across the cancer care continuum from diagnosis through treatment, survivorship, and end-of-life. This article describes the characteristics and determinants of ED visits, as well as challenges in the effort to define preventable ED visits in this population.

**Findings:**

The most recent population-based estimates suggest 4% of all ED visits are cancer-related and roughly two thirds of these ED visits result in hospitalization—a 4-fold higher ED hospitalization rate than the general population. Approximately 44% of cancer patients visit the ED within 1 year of diagnosis, and more often have repeat ED visits within a short time frame, though there is substantial variability across cancer types. Similar patterns of cancer-related ED use are observed internationally across a range of different national payment and health system settings. ED use for patients with cancer likely reflects a complex interaction of individual and contextual factors—including provider behavior, health system characteristics, and health policies—that warrants greater attention in the literature.

**Conclusions:**

Given the amount and complexity of cancer care delivered in the emergency setting, future research is recommended to examine specific symptoms associated with cancer-related ED visits, the contextual determinants of ED use, and definitions of preventable ED use specific to patients with cancer.

## Background

Globally, clinicians and policymakers are increasingly focused on improving the quality of cancer care by optimizing health care delivery [1–3]. Current estimates project dramatic increases in new cancer diagnoses, cancer survivorship, and costs of care [4–6]. Furthermore, as global cancer incidence increases, the burden of cancer care is expected to fall increasingly on lower-income countries [7]. With improving cancer treatments and survival, the primary and specialty care needs of cancer survivors will have substantial implications for health systems worldwide.

Approximately 4% of all adult emergency department (ED) visits in the US are for cancer-related complaints [8, 9]. Although this may not seem to be a high proportion, it represents a substantial number of visits amongst the extraordinary variety of conditions treated in EDs. These visits tend to be of higher triage acuity and severity, especially among patients with concurrent co-morbid conditions and among older adults with complex care needs [8, 10]. Furthermore, this rate is comparable to the proportion of visits related to congestive heart failure (4%), chronic kidney disease (3.5%), cerebrovascular disease including strokes (3.7%), and highly prevalent chronic conditions such as diabetes (6%) [9].

Reports from Australia, the United Kingdom (UK), Brazil, and South Korea highlight similar concerns about the growing number of cancer patients and the increasing burden on EDs to manage disease- and treatment-related acute care [11–14]. A 2017 National Health Service report from the UK emphasized dramatic increases in ED presentations related to cancer and concomitantly high rates of inpatient admission—often associated with poor patient experience, inadequate communication, and fragmented care coordination [15].

**Fig. 1:**
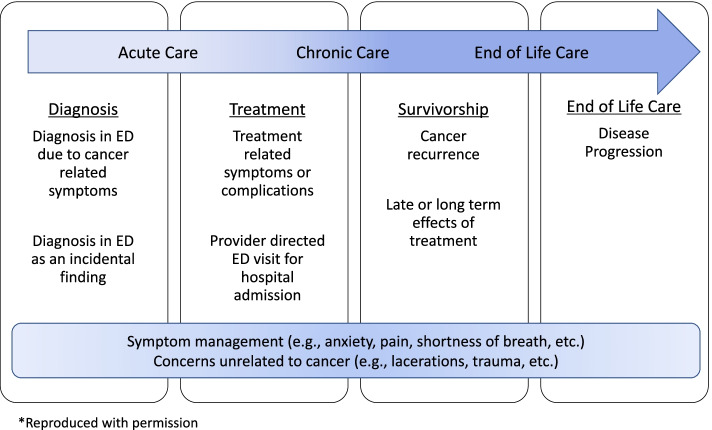
Emergency department visits across the cancer care trajectory [16]

Patients with cancer present to the ED across the cancer survivorship continuum: at diagnosis, through treatment, post-treatment periods, and at the end-of-life (Fig. 1) [16]. Their reasons for ED visits range from time-sensitive emergencies to use of the ED as an entry point for a hospital admission. Notably, patients with cancer often incur multiple visits [17, 18]. Patients with cancer may also present to the ED for conditions seemingly unrelated to their cancer (e.g., motor vehicle collisions, musculoskeletal injuries), but care for these patients is potentially complicated by cancer and its treatment [16, 19].

ED use for patients with cancer reflects complex interactions between individual factors (e.g., insurance status, ability to use services), provider factors (e.g., knowledge, skills, communication, referrals, and access to specialists), health system factors (e.g., bed capacity), and policy factors (e.g., availability of social care) [20]. While the number of ED visits per capita remained consistent between 2009 and 2018, costs have increased dramatically and crowding remains a persistent problem [21, 22]. EDs have continued to attract scrutiny as an inefficient and fragmentary site of care. This has led to an interest in defining “avoidable” or “preventable” ED visits, though research on this is still in the early stages [14, 23–26]. Additionally, policy makers and practitioners have focused on non-urgent ED visits, reflecting patient desires to avoid ED care and its subsequent disruptions to ongoing cancer treatment [27].

Despite growing interest, patterns of ED use among patients with cancer are still poorly understood. Variations in study methodology and heterogeneity in the mix of studied cancer types make comprehensive descriptions of ED use challenging. This article summarizes the current evidence about what is known concerning ED use by adult patients with cancer, specifically, the distribution of visits (i.e., frequency, incidence, and disposition), determinants of use, and the preventability of visits. Descriptions of patterns of ED utilization among pediatric oncology patients are described elsewhere [28–30]. Similarly, descriptions of specific interventions to reduce ED use and the impact of palliative care in this population are not explored in this review. We present the US-based literature first and then add comparisons from international reports.

### Distribution of ED visits among patients with cancer

#### Data sources

In the US, there are few robust data sources linking important clinical cancer details and comprehensive health care utilization. Nationally representative administrative data sets capturing complete utilization do not reliably capture incident cancer diagnoses, stage, treatment information, or complete co-morbidity data (e.g., Medical Expenditure Panel Survey [31], Nationwide Emergency Department Sample [32], National Hospital Ambulatory Medical Care Survey [33]). These datasets often rely on a clinician coding a cancer diagnosis during a patient encounter or reporting patient survey responses with little detail, so it is difficult to ascertain how recently a patient was diagnosed or whether treatment is ongoing.

Local and regional cancer registries collect specific cancer and initial treatment information, but often have limited health care utilization data, including ED visits [34]. The Surveillance Epidemiology and End Results (SEER) program includes population-based information about cancer incidence and survival but does not provide related health services data [35]. Researchers can link these registries to utilization data—most notably SEER linked to Medicare insurance claims; however, this is limited only to subsegments of insured cancer patients. Some state-level all-payer claims datasets can be linked to statewide cancer registries, but these datasets may not contain claims from all insurers or the uninsured, and it is often difficult to longitudinally link patients as they change insurance plans over time [36]. Therefore, the incidence and frequency of cancer patients visiting EDs in the US are difficult to conclusively ascertain [24].

#### Estimates

##### Cancer-related ED visits

Despite data limitatons, the most recent population-based estimates suggest that between 2006 and 2014 over 4% of all US-based ED visits were cancer-related [8, 37]. Geographically, patterns of ED use are consistent across regions of the US (Northeast, Midwest, South, West) [37]. The number of ED visits varies by the  primary cancer diagnosis, and there are large numbers of ED visits comprised of patients with breast, lung, prostate, and colon cancer because they are relatively common cancers. The top cancers associated with ED visits include lung 10–27% [8, 17, 38–40], breast 6–15% [8, 17, 38–40], colon 6–12% [8, 17, 38–40], prostate 5–11% [8, 17, 38–40], multiple cancers 10% [8], and female reproductive or genital 6–7% [8, 17, 39]. These ED visit level reports largely reflect the population prevalence of cancer survivors and may not account for patient-specific information (diagnosis and treatment data) nor account for multiple visits by the same patient.

##### Incidence of ED use among patients with cancer

Among all patients with cancer, the presence of any ED visit and the distribution and timing of ED visits is difficult to describe, in part because estimates depend on variable precipitating triggering events. ED visits are measured within a particular time span (e.g., from diagnosis or from treatment, such as surgery, radiation, or chemotherapy), but the time spans vary greatly [24]. For example, one study of breast cancer patients receiving a mastectomy reported that 3% of the sample had an ED visit within 30 days of surgery [41] while 11% of high-risk patients with head/neck cancer receiving radiation had an ED visit during treatment or within 90 days of treatment completion [42].

Using a standardized 30-day visit rate, studies that examined postsurgical periods reported 2–12% of the sample visited an ED within 30 days after surgery and a study that evaluated a post-chemotherapy time frame demonstrated 5% of the sample visited the ED [24]. Estimates provided by the population-based studies tend to report higher ED use than those that focus on smaller single-setting samples [24].

Time from diagnosis offers a consistent measure to examine ED use by patients with cancer across cancer types, although it is important to consider the factors that impact time to diagnosis. We summarize some estimates at 30, 180, and 365 days from diagnosis in Table 1 [17, 18, 43, 44]. Due to differences in diagnostic and treatment patterns, the incidence of ED visits varies by cancer type. It is important to distinguish between the patient level and visit level estimates. This table shows that patients with certain cancers, including lung, colon, and pancreas, are more likely to have an ED visit.

**Table 1:** Cumulative percentage of cancer patients with at least one ED visit by time from diagnosis [17]

Cancer type	Time from diagnosis		
	30 days	180 days	365 days
All	17	35, 44–69 [44]	44
Bladder	21	44	54
Brain	39	60	68
Breast	5	22	31, 15–21 [43]
Colon	20	41	49, 55 [19]
Digestive	26	54	63
Endocrine	7	19	25
Eye	6	18	26
Gynecological	17	36	44
Hodgkin lymphoma	18	43	46
Ill-defined/unknown	36	53	57
Leukemia	26	45	53
Liver	29	54	63
Lung	30	55	64
Male genital (non-prostate)	16	28	36
Melanoma	5	14	22
Myeloma	28	53	63
Non-Hodgkin lymphoma	22	44	51
Oral	12	39	48
Other	20	42	53
Pancreas	37	62	69
Prostate	6	17	25
Respiratory (non-lung)	18	43	52
Stomach	27	55	63
Urinary	21	39	47

These estimates are from four population-based studies that provide data on ED visits by cancer patients and time from diagnosis. Unless specified, data are derived from California state-based data from 2009 to 2010 [17]

##### Multiple visits

A large proportion of cancer patients have multiple ED visits [17, 18]. A California population-based study reported that 20% of patients with cancer had one ED visit, 8% had two visits, and 7% had three or more visits within 180 days of diagnosis. Among those with at least one ED visit, 44% had two or more visits and 21% had 3 or more visits [17]. These rates of multiple visits are substantially higher than in the general patient population where only 6.5% have two or more ED visits in a year [45]. These ED visits are made to a wide range of facilities: patients who are being treated at one health system may only present to that system’s ED for roughly one in three visits, underscoring the importance of the data limitations we discussed above [46].

##### Disposition

While inpatient admission rates vary by cancer type, patients with cancer who visit the ED have higher rates of admission (49–63%) and are four to five times as likely to be admitted than non-cancer patients (12%) [8, 9, 17, 37, 38, 47]. Patients with cancer are more likely to be admitted to a progressive care or intensive care unit (11%) compared to the general population (2%) [9, 10]. Between 34 and 49% are discharged home [8, 10, 17] and approximately, 4–5% of the remaining patients are transferred to another facility, die during the ED visits, or leave before physican evaluation or against medical advice [10, 48].

### Determinants of ED use among cancer patients

The Andersen Behavioral Model of Health Services Use provides a framework to understand the determinants of ED use among cancer patients [49]. In this model, health service use is determined by the interaction between societal, individual (predisposing characteristics, enabling resources, and illness severity factors), and health system characteristics. The individual-level factors associated with ED use tend to be similar to the general patient literature--including chronic conditions such as diabetes or other sub-populations such as older adults (Table 2) [50, 51].

**Table 2:** Individual determinant of ED use [49, 52]

Description	Factors associated with general ED use
Predisposing factors *Demographic, social structures (education, religion), and individual beliefs*	▪ Age: Adults age 65 years older age ▪ Sex: Females compared to males ▪ Rates are lower among those living in the West compared to other regions
Enabling factors *Income, health insurance, access to regular care, area if residence*	▪ Private insurance or Medicaid compared to Medicare or no insurance ▪ Residence in low-income areas
Illness severity factors *Perceived (disability, symptoms, general state) and Evaluated (diagnosis)*	▪ Diagnosis: abdominal pain, chest pain, back problems, urinary tract infections, or skin infections

The predisposing, enabling, and illness severity factors associated with ED visits among patients with cancer vary by cohort definition (e.g., by cancer type or area of residence), treatment, complications of interest, and time frame studied. Among the US studies, there is heterogeneity in the time period examined (generally within the first year after diagnosis but as short as within 30 days of treatment), the number of participants (*n*=220 to 89,311), and cancers included [18, 24, 53]. Some studies focus on older adults, precluding the ability to examine age as a determinant of ED use. Many studies focus solely on one or more of the top four most prevalent cancers (i.e., prostate, breast, lung, and colorectal) [24].

#### Predisposing factors

Similar to the general population, for patients with cancer, predisposing factors of non-white [18] and African American [50, 54, 55] race/ethnicity, and older age [18, 50] were associated with more ED use, compared with white, non-Hispanic race/ethnicity, and younger age. Being male compared to female resulted in higher rates of ED use [56, 57]. Additionally, ED use before a cancer diagnosis has been found to be highly predictive of ED utilization after a cancer diagnosis [55, 58]. 

####  Enabling factors

Enabling factors associated with higher ED use for patients with cancer also reflect the same factors as for the general population: higher ED use is associated with urban dwellers compared to rural residents [50], Medicaid or uninsured status [55], and changes to insurance eligibility or cost-sharing structure [44, 59]. Unmarried compared to married patients and low neighborhood median incomes are also associated with higher ED use [50].

#### Illness severity factors

Operational definitions of illness severity factors for ED care include reasons for visit, symptoms, chief complaints, and coded diagnoses, and they vary across healthcare settings and studies. Ultimately, the underlying symptoms or diagnoses associated with ED visits are illness severity-related determinants of ED use. Cancer patients present to the ED with a variety of complaints but tend to have symptoms or diagnoses related to pain, respiratory, gastrointestinal, cardiac, and infectious concerns.

A systematic review of symptoms experienced by cancer patients visiting the ED identified 28 reported symptoms, including psychological (such as anxiety), gastrointestinal, neurological, respiratory, dermatological, urological, pain, fever and infection, edema, bleeding, fatigue, and altered nutritional status [60]. The primary reason for visit or chief complaint, as opposed to coded diagnoses, tends to be related to pain, respiratory distress, fever, and gastrointestinal issues [37, 38, 47].

In contrast, some recent population-based studies use only coded diagnoses to describe the reason for an ED visit [8, 17, 23]. A national sample of cancer-related ED visits from 2006 to 2012 reported pneumonia as the most common diagnosis, accounting for 4.5% of cancer-related visits [8]. Other diagnoses that each represented between 3 and 4% of visits were nonspecific chest pain, urinary tract infections, septicemia, and chronic obstructive pulmonary disease [8]. Abdominal pain, fluid and electrolyte disorders, congestive heart failure, cardiac dysrhythmia, and intestinal obstruction without hernia each represented between 2 and 3% of the ED visits [8].

This discrepancy between reasons for ED visit is likely explained by the fact that ED diagnosis codes are entered after complaints have been differentiated by thorough clinical evaluation. For example, a patient presenting with chest pain could ultimately be diagnosed with a wide range of diagnoses as well as non-specific chest pain [61]. This, in part, explains the differences in “reasons for ED visit” between studies that use presenting complaints and those that use diagnosis codes.

ED visit diagnoses also vary by disposition [17]. Yet, regardless of visit disposition, the top 10 diagnoses cumulatively account for less than 40% of all visits, underscoring the variability in precipitating factors for ED visits among cancer patients [8, 17]. Furthermore, how specific codes are grouped into clinically meaningful categories can vary according to the scenario studied, such as surgical complications versus chemotherapy-related complications.

Finally, other studies have reported that patients with a greater number of comorbidities [18, 59, 62], a diagnosis of depression compared to no depression [63], various combinations of cancer treatments—chemotherapy and/or radiation therapy to specific targets [18, 50, 54, 55, 59, 64, 65], more severe symptoms [62], longer delays to initiation of treatment [54], and those close to the end of life (less than 1 year of survival after diagnosis) [56, 66] all had higher rates of ED use. Although cancer stage is considered an illness severity factor that increases ED use, evidence on this is somewhat mixed [24, 55]. Given the wide variety in study methodology, it is difficult to summarize ED visits in relation to specific cancer treatments and phases of cancer care despite the clinical importance of these factors.

#### Health system factors

Although there are often clinician phone line resources available for cancer patients, recent research suggests that patients with cancer do not often call before going to the ED, and even when they call, they are frequently directed to the ED by the clinician [27, 46]. A potential explanation of the underuse of these phone-based triage resources is suggested by qualitative work finding that patients feel guilty for bothering providers [27]. While a common explanation of non-urgent ED use is that other outpatient alternatives were closed, at least two population-based US studies have found that roughly half of ED arrivals occur during business hours [38, 48].

### Preventability of ED visits among cancer patients

One of the primary goals of examining ED visits is to identify care that could have been prevented or more optimally delivered in an alternate setting. Despite great attention to preventable ED use, widely used measures rely on diagnosis codes only, and there is little consensus on measures specifically for patients with cancer [24]. The existing validated diagnosis code-based measures to define preventable ED visits include cancer patients, but are not cancer-specific [23, 24]: for instance, febrile neutropenia is not classified by these measures [23, 24].

We outline the details of larger efforts to define preventable ED use in patients with cancer in Table 3. In 2016, the Centers for Medicare & Medicaid Services (CMS) established a quality metric that tallies anemia, dehydration, diarrhea, emesis, fever, nausea, neutropenia, pain, pneumonia, or sepsis within 30 days of outpatient chemotherapy or immunotherapy administration [26]. Using claims data, Panattoni et al. quantified the proportion of ED visits defined as preventable according to this definition, as well as from a patient-reported outcome symptom tracking tool, identifying 18 diagnoses representing potentially preventable ED visits in the year after chemotherapy, radiation or both, that accounted for 40% of the primary diagnoses of ED visits, up to 63% if including all coding fields in the visit [23].

**Table 3:** Criteria for identifying preventable ED visits among patients with cancer

Panattoni, et al. [23]^a^	CMS [26]^b^	Roy et al. [67]^c^
Anemia	Anemia	
Appetite loss		Appetite loss
Constipation		Constipation
Cough		Cough
Dehydration	Dehydration	Dehydration
Diarrhea	Diarrhea	Diarrhea
Dyspnea		Dyspnea
Dysuria		Dysuria
Emesis	Emesis	Emesis
Fatigue		Fatigue
Fever	Fever	
Flushing		Hot flashes
Nausea	Nausea	
Neuropathy		Neuropathy
Neutropenia	Neutropenia	
Pain	Pain	Pain
Pneumonia	Pneumonia	
Sepsis	Sepsis	
		Vomiting

^a^Diagnosis

^b^Within 30 days of outpatient chemotherapy treatment

^c^Complaints (not diagnosis)

A closer examination of these diagnosis code-based definitions of preventable raises questions. Conditions such as sepsis, pneumonia, or dyspnea may represent clinically significant events that frequently justify ED visit and hospital admission and are difficult to imagine how to prevent. Given that the diagnoses described in Table 3 may be present in over half of cancer-related ED visits but these patients might actually have serious life-threatening conditions, risk stratification methodologies are needed to better distinguish which ED visits could be safely be prevented [23, 39]. More careful clinician chart reviews of ED visits find a lower proportion of potentially preventable visits—10–25% of ED visits in one site [67] and roughly 20% of hospitalizations from another [68]. Finally, all of these determinations are made based on clinician expertise and opinion, yet it remains unclear how even well-equipped health systems and clinical settings could prevent this purportedly preventable care.

Patients who obtain their initial cancer diagnosis in the ED, roughly 12–32% of patients with cancer, represent an ongoing clinical challenge [69–72]. The symptoms leading to ED visits may be either directly related to the cancer, or cancers may be incidental findings during evaluation. In either circumstance, it is difficult to establish whether these visits were potentially avoidable based on diagnosis codes and even more difficult to determine if limited access to primary care or cancer screenings contributed to a delayed diagnosis in the ED.

### International perspectives

#### Frequency of ED use

ED use by cancer patients outside the US is also characterized by high incidence and frequency, across a range of payment and medical system structures [73–79]. For example, in Australia 40% of cancer patients visited the ED at least once in the year following diagnosis, 63% of these ED visitors had multiple visits, and 2.4% of all ED visits were made by cancer patients [74, 75]. Studies from other countries report high frequencies of patients with multiple ED visits as well [74, 77, 80, 81]. For instance, in Canadian breast cancer patients receiving chemotherapy, nearly 37% of those with at least one ED visit within 30 days of treatment had multiple visits [77]. Yet, direct inter-country comparisons are difficult due to differential cancer and comorbidity burdens, health system structures, and treatment patterns.

#### Determinants of ED use

A range of international studies describes a similar ecology of reasons for ED visits. Similar to the US, studies from Brazil, Australia, the UK, Canada, and Taiwan identify the most common complaints to be abdominal pain, back pain, dyspnea, weakness/fatigue, infection, fever (neutropenic and non- neutropenic), nausea/vomiting, drug reaction, surgical site issues, shortness of breath, and process of care issues [76, 78, 81, 82].

Studies from the UK, Australia, and Canada find that ED visit likelihood varies by age and gender within certain cancer types: head and neck, upper gastrointestinal, colorectal, lung, breast, and women diagnosed with cancers of the bladder [13, 69, 75]. Other predictors of higher ED use include low socioeconomic status (for most cancers), having comorbidities, surgery in the prior 45 days of surgery, mastectomy versus lumpectomy, operation before definitive oncologic control, lower institutional volume, polypharmacy, benzodiazepine use, anticoagulant use, cardiovascular disease, diabetes, and past hospitalization [69, 75, 78].

#### International perspective on preventable ED use

Despite advantages in data centralization, international efforts to define potentially preventable ED visits have met similar challenges to those in the US. Some reports from nationalized health care systems focus on cancer diagnoses in the ED as a particular subset of preventable ED use [71, 83]. Other studies from Canada, France, and South Korea all use different definitions and methods to describe preventable visits [12, 84, 85]. These definitions of avoidable include care related to device problems, constipation, repeated prescriptions, follow-up visits, or laboratory examinations; the need for a medical exam within 24 h; or a problem that could be resolved at an outpatient clinic, or over the telephone, while considering visits due to hospice referral, chemotherapy or radiotherapy, or surgery as unavoidable [12, 84, 85]. Using these varied definitions between 7 and 60% of visits may be classified as potentially avoidable.

Finally, we note that the international reports almost exclusively come from developed nations with robust health care systems. Some of this may be explained by the larger structural differences in the organization of acute and cancer care in less developed nations. With the global cancer burden shifting to developing countries, the ability to address acute care needs remains an important issue.

## Conclusion

One of the greatest challenges to providing high-quality cancer care worldwide is the management of unplanned acute care during cancer treatment [86]. The need for well-coordinated end-of-life and palliative care is well established, but acute care, ED visits, and hospitalizations still increase towards the end of life [3]. While a few select health systems have developed oncology-specific EDs, urgent care, or walk-in clinics to address unscheduled concerns, the majority of patients receive cancer treatment in less concentrated community-based settings and may rely on general EDs for their unplanned acute care needs [3]. The literature is only beginning to address the impacts of modern immunotherapies, further complicating our ability to anticipate urgent and emergent acute care needs. While cancer patients have a higher risk of hospital admission and death, other important ED-specific outcomes could be explored, taking into account treatment regimens, specific symptoms, and patient experiences [19].

Understanding ED use by patients with cancer requires careful clinical examination of both the frequency and reasons for visits by cancer patients as well as assessment of upstream drivers. Such research would better prepare health providers and systems to develop innovations that address optimizing ED use, and allow partnering with policy makers to refine definitions of preventable ED use in this population. This information is essential to implementing and disseminating successful oncology care innovations which may include tele-medicine, enhanced symptom management support systems, care pathways, oncology-specific EDs, and other ED alternatives. Of particular interest may be multi-level interventions that address at least three levels of the multi-layered health care system (e.g., individual, provider team, organization, community, or health policy) [87].

Determinants of ED use include a complex constellation of predisposing, enabling, and illness severity factors, with most studied at the individual patient level. However, nearly all are immutable demographic or clinical characteristics that are not clear targets for direct or indirect intervention. An actionable list of intervenable factors associated with increased individual ED use is lacking. More broadly, little is known about the contextual determinants of ED use—including provider, health system, and policy factors—in part because of limitations in extant data. Future research and data investments should be targeted to address these gaps. Likewise, while the impact of the COVID-19 pandemic is still evolving, it is reasonable to assume that there will be delays in cancer diagnosis and complications that will inevitably impact the care of these patients in emergent and urgent settings [88].

The variability in defining avoidable ED visits in countries with robust national health systems and centralized data highlights the complexity of describing ED care needs for the cancer patient population. However, conservative estimates suggest that there is substantial room for improvement. A potentially fruitful direction may be to develop a deeper understanding of some of the most frequent reasons for visits: pain, dyspnea, nausea/vomiting, and concerns for bacterial infection [39]. Lastly, the very definition of avoidable ED visits from the clinician standpoint is also unclear and may be context-dependent. We suggest a shift towards increasing the patient-centeredness of the provision of cancer care, both scheduled and unscheduled, to provide a concrete direction for improving the quality of unplanned acute care.

## Data Availability

Not applicable.

## Abbreviations

ED: Emergency department
US: United States
SEER: Surveillance Epidemiology and End Results
CMS: Centers for Medicare & Medicaid Services
